# *In Vivo* Comparison of 23- and 25-Gauge Sutureless Vitrectomy Incision Architecture Using Spectral Domain Optical Coherence Tomography

**DOI:** 10.1155/2013/347801

**Published:** 2013-03-04

**Authors:** Anderson Teixeira, Flavio A. Rezende, Camila Salaroli, Nonato Souza, Benedito Antonio Sousa, Norma Allemann

**Affiliations:** ^1^Teixeira Oftalmologia, SDS Bloco D No 27, Sala 306, 70392-901 Brasília, DF, Brazil; ^2^Department of Ophthalmology, Universidade Federal de São Paulo, São Paulo, SP, Brazil; ^3^Department of Ophthalmology, Universidade Católica de Brasília, Brasília, DF, Brazil; ^4^Department of Ophthalmology, Hôpital Maisonneuve-Rosemont, Université de Montréal, Montréal, QC, Canada; ^5^Department of Ophthalmology, Pontifícia Universidade Católica, Rio de Janeiro, RJ, Brazil; ^6^Department of Ophthalmology, University of Illinois at Chicago, Chicago, IL, USA

## Abstract

*Purpose*. To investigate the *in vivo* incision architecture using spectral domain optical coherence tomography (SD-OCT) in 23-gauge and 25-gauge transconjunctival sutureless *pars plana* vitrectomy (TSPPV). *Methods*. A prospective observational study of 22 eyes of 22 patients that underwent three-port 25-gauge (10 eyes) or 23-gauge (12 eyes) TSPPV was performed. The three sclerotomies sites in each eye were analyzed by Corneal Adapter Model (CAM) RTVue SD-OCT (Optovue Inc., Fremont, CA, USA) with wound cross-section images (longitudinal and transversal) on days 1, 7, and 30 postoperatively. Transversal and longitudinal length, location, angle between the conjunctival surface tangent and the incision plane, and architecture deformations were evaluated. *Results*. All patients (22 eyes) completed the study and surgeries lasted less than 60 minutes. All wounds were obliquely performed, 23-gauge mean angle was 23 ± 5°, and 25-gauge angule was 21 ± 4°. Twenty-three-gauge sclerotomy transversal mean length was 1122 ± 242 **μ**m and 25-gauge transversal sclerotomy mean length was 977 ± 174 **μ**m; 23-gauge longitudinal mean length was 363 ± 42 **μ**m and 25-gauge longitudinal sclerotomy mean length was 234 ±19 **μ**m; 23-gauge open wound thickness mean was 61 ± 28 **μ**m and 25-gauge open wound thickness mean was 22 ± 6 **μ**m. All results were statistically significant (*P* < 0.05). No vitreous incarceration or silicone oil residue was observed in incision sites with both gauges. *Conclusions*. The 23-gauge and 25-gauge architectural wound constructions were well visualized using CAM SD-OCT. Statistical differences between the two gauges were observed throughout the study period.

## 1. Introduction

Transconjunctival sutureless *pars plana* vitrectomy (TSPPV) has considerably transformed the outlook of patient management in the field of retina and vitreous surgery since when described in 2002 [1, 2]. At first, the cannulas were introduced straight through the sclera at the sclerotomy sites in a perpendicular fashion. However, with this technique questions have been raised regarding to the true self-sealing characteristics of some of these TSPPV, with reports of increased hypotony and endophthalmitis rates associated [3–9]. The creation of the beveled opposed to a straight sclerotomy incision to prevent leakage through these sutureless wounds was a greater advantage for the procedure [10–14]. But the limitations of the instruments, fluidics, extensive flexibility of the instruments, and poor illumination caused by a small lumen 0.5 mm caliber reduced the use of 25-gauge TSPPV, even with advantages over the traditional techniques such as reduced surgical trauma, improved postoperative comfort, faster visual recovery, shorter operating times, and reduced postoperative astigmatism [2, 15, 16]. This procedure became increasingly most commonly used when 23-gauge TSPPV was introduced to overcome the limitations of 25-ga describe above [17–20].

However, there still exists debate of the self-sealing characteristics of TSPPV with respect to the best wound architecture construction. Recently, ultrasound biomicroscopy (UBM) [21] and time-domain optical coherence tomography (OCT) [22–24] have been used to evaluate wound architecture *in vivo*. The aim of this study was, therefore, to use the high-resolution anterior segment (AS) spectral domain OCT (SD-OCT) to investigate the wound architecture of both 23-ga and 25-gauge TSPPV in order to gain further insight into the sclerotomy wound construction.

## 2. Materials and Methods

Twenty-two consecutive patients (minimum age: 18 years old) have undergone primary TSPPV 23-gauge or 25-gauge using one-step Alcon/Grieshaber trocar cannula set (Alcon/Grieshaber, Alcon Laboratories, Inc., Fort Worth, TX, USA) and Accurus 400 VS vitreous machine (Alcon Laboratories, Inc., Fort Worth, TX, USA). In this study we used the new generation of the trocar cannula set that is composed of trocar and metal cannula, different from the oldest generation that is composed of polymer cannula for the 25-gauge set. The surgical parameters were for 23-gauge: 1800–2500 cuts per minute (CPM) and 200–400 mmHg of vacuum—using 3D parameter; and 25-gauge: 1800–2500 CPM and 400–600 mmHg of vacuum—using 3D parameter. Study participants were recruited from the clinical practices by the coauthors AT and CS according to a prospective study protocol approved by the institutional review board. Informed consent was obtained and documented for every participant. The study adhered to the tenets of Declaration of Helsinki. The eye was randomly selected for each gauge, and all TSPPV surgeries were performed by the same surgeon (AT), within less than 60 minutes. Exclusion criteria included history of previous *pars plana* vitrectomy surgery.

Demographics of patients, diagnoses, and type of procedures performed were also recorded. Anatomical outcomes and any postoperative complications, especially sclerotomy-related ones, were specifically evaluated. A spectral-domain OCT system (RTVue, Optovue Inc., Fremont, CA) with a corneal adaptor module (CAM) was used to image the sclerotomies on postoperative days 1, 7, and 30. A crossline scan pattern was used to measure the scleral incisions positioning the caliper mark perpendicularly to the limbus.

**Figure 1:**
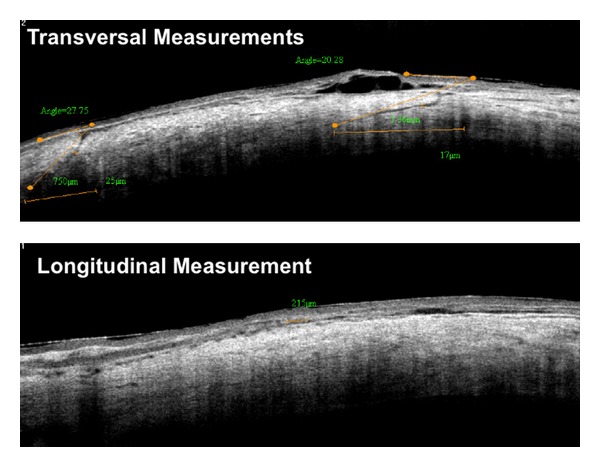
Images of the 25-gauge sclerotomies on postoperative. A cross line scan parameters of transverse and longitudinal measurements illustrated with a diagrams.

A conventional three-port vitrectomy was performed with a straight angled incision of 20 degrees to 30 degrees in all patients; the wound locations were superotemporal, superonasal, and inferotemporal in all eyes. Measurements of the transversal and longitudinal incision length and of the angle formed between the conjunctival surface tangent and the incision plane were taken; besides, information about location and architecture deformations was collected (Figure 1). SAS V9.1 programming language (SAS Institute Inc., Cary, NC, USA) was used for statistical analyses. Accepted level of significance for all tests was a *P* value of less than 0.05.

## 3. Results

Average patient age at time of surgery was 65.1 years ± 15.1 (range 36–83 years). Ten patients were men. Thirty 25-gauge sclerotomies of ten eyes and thirty-six 23-gauge sclerotomies of 12 eyes were analyzed. Indications for surgery were epiretinal membrane (4), rhegmatogenous retinal detachment (RD) (4), tractional RD (8), and macular hole (6). Combined phacoemulsification and IOL implantation through clear corneal incision were performed in 2 eyes with RD. All cataract incisions were performed after the infusion trocar was placed. 10–0 nylon suture was used at the corneal incision to prevent anterior chamber collapse during the cannula insertion. There was no failure in cannula insertion due to hypotony or anterior chamber collapse. In two patients with RD a silicone intraocular injection after vitrectomy (one for each gauge) was necessary. All patients with RD underwent TSPPV without scleral buckle.

No intraoperative suture placement was necessary and no patient had hypotony on the first postoperative day. The IOP was normal in all postoperative visits (range: 12 to 22 mmHg).

**Figure 2:**
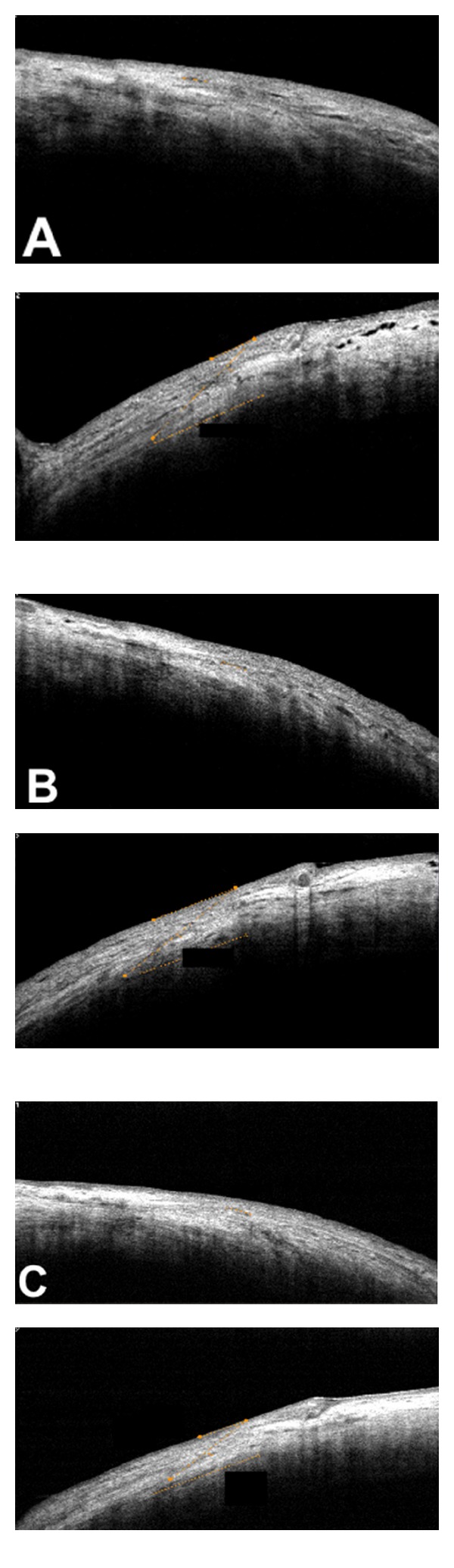
Spectral domain OCT images (RTVue with a corneal adaptor module-CAM from Optovue Inc., Fremont, CA) at the postoperative period of 23-gauge sutureless pars plana vitrectomy. (A) Longitudinal (upper) and transversal (bottom) length measurement at the sclerotomy incision, on postoperative day 1. (B) Longitudinal (upper) and transversal (bottom) length measurement at the sclerotomy incision, on postoperative day 7. (C) Longitudinal (upper) and transversal (bottom) length measurement at the sclerotomy incision, on postoperative day 30.

**Figure 3:**
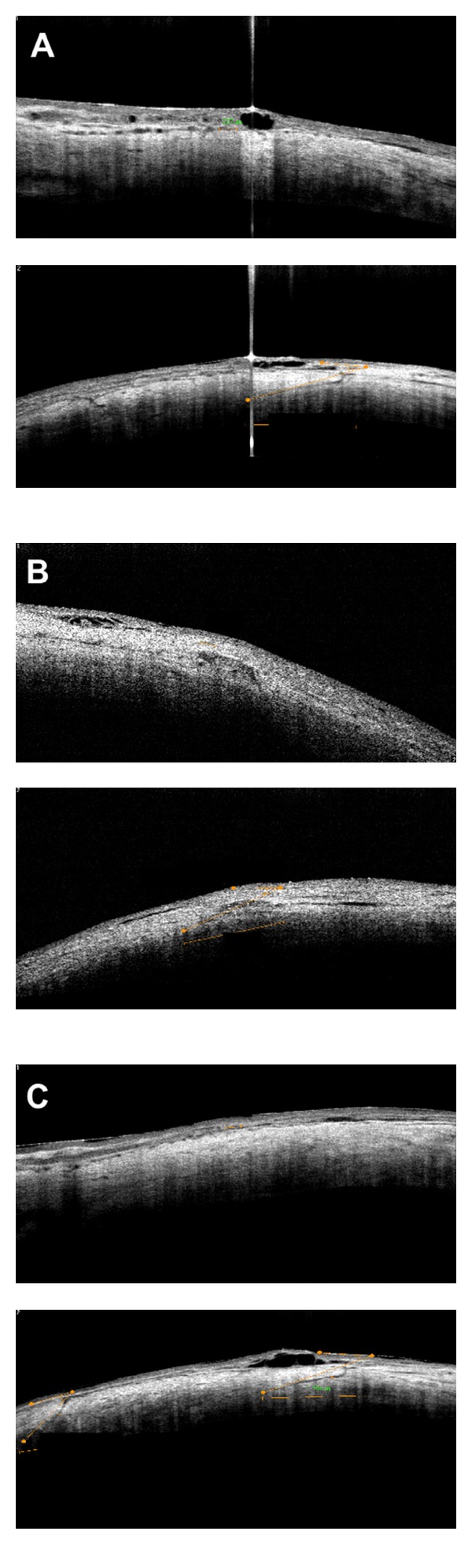
Spectral domain OCT images (Optovue with Cornea Camera) at the postoperative period of 25-gauge sutureless pars plana vitrectomy. (A) Longitudinal (upper) and transversal (bottom) length measurement at the sclerotomy incision, on postoperative day 1. (B) Longitudinal (upper) and transversal (bottom) length measurement at the sclerotomy incision, on postoperative day 7. (C) Longitudinal (upper) and transversal (bottom) length measurement at the sclerotomy incision, on postoperative day 30.

All three incisions of each eye were evaluated using SD-OCT to define incision on postoperative days 1, 7, and 30. Transversal and longitudinal length, location, angle formed between the conjunctival surface tangent and the incision plane, and architecture deformations were evaluated. All wounds were obliquely performed, 23-gauge mean incision angle was 23 ± 5° (range: 37 to 5 degrees), and in 25-gauge, 21 ± 4° (range: 31 to 4 degrees). Mean transversal sclerotomy length was 1122 ± 242 *μ*m with 23-gauge (range: 1781 to 283) and 977 ± 174 *μ*m (range: 1650 to 174) with 25-gauge. Mean longitudinal sclerotomy length was 363 ± 42 *μ*m (range: 543 to 42) with 23-gauge, and 234 ± 19 *μ*m (range: 265 to 19) with 25-gauge. Mean open wound thickness was 61 ± 28 *μ*m (range: 543 to 42) with 23-gauge and 22 ± 6 *μ*m (range: 48 to 6) with 25-gauge. All results were statistically significant (*P* < 0.05). No vitreous incarceration or silicone oil residue was observed in incision sites with both gauges (Figures 2 and 3), and no architectural structure difference was observed comparing macular to RD cases. Tables 1 and 2 describe transversal and longitudinal results for all gauges.

## 4. Discussion

Chen first described sutureless self-sealing sclerotomies in 1996 [25]. After that, other authors have described their experience and technique modification [1, 2, 17, 26]. Twenty-three and 25-gauge tunnel incision cannulas require no suture because of a reduced diameter, 0.5 mm [1] and 0.72 mm [17], respectively, placed obliquely to the scleral surface. The longitudinal length of the sclerotomy was around 50% of the lumen diameter of the gauges. On postoperative days 1 and 30, the incision length showed low variance, suggesting that the scleral fibers were partially cut and partially dissected without damage, suggesting no variation during those days (Table 1).

Imaging exams, such as UBM [21] and AS-OCT, serve as a tool to produce clear cross-sectional images of the *pars plana*, including vitreous base. These regions are difficult to examine adequately using lenses and/or slit lamp, and it may be useful to locate the sclerotomy site [27]. Boker and Spitznas reported the use of UBM to examine the sclerotomy site after PPV [28]. In a series of 20 eyes of 19 patients, the entry sites were identified by UBM, and the authors concluded that UBM was useful in determining possible sources of complications after vitrectomy and could give further insight into the interactions between *pars plana* sclerotomy and anterior fibrovascular proliferation [29]. The major shortcoming of UBM is that the direct pressure of the open immersion cup on the incisions could alter the observed architecture and increase the potential risk of endophthalmitis, especially in the early postoperative period [22]. The risk of compression with the UBM exam can possibly be reduced using the ClearScan bag/balloon available.

Some authors demonstrated the importance of the oblique incisions *in vitro* studies where angled incisions were superior at histopathology and in AS-OCT [14, 23, 30]. Taban et al. using time domain AS-OCT imaging demonstrated that 23-gauge sutureless vitrectomy performed using oblique (angled) incisions provided adequate wound closure evident even on postoperative day one [23].

Anterior segment OCT is a noncontact and noninvasive imaging exam and considered for serial examination. Time domain AS-OCT is well suited to examine sutureless vitrectomy incisions in the immediate postoperative period, with adequate penetration depth and low resolution [22]. SD-OCT images obtained not only clearly showed the profile of the incisions with a high resolution that allowed accurate measurement of parameters such as incision length and angle, but also identified the fine architectural features identified by the authors as wound opening thickness. SD-OCT has a limitation related to depth.

In the present study, we were able to identify the three sclerotomy sites with SD-OCT using the corneal camera. All wounds were obliquely built with a safety angle of entrance, longitudinal diameter was close to 50% of the cannula lumen, and transversal wound length (distance between the external and internal scleral limits) was around 1 mm. There was an adequate wound closure with a hyporeflective signal corresponding to an open wound thick scar. All results were statistically significant showing difference between the wound architecture comparing sutureless vitrectomy with 23-gauge and 25-gauge.

## Figures and Tables

**Table 1:** Transversal length of sclerotomy incisions after transconjunctival sutureless pars plana vitrectomy performed with 23-gauge and 25-gauge measured with SD-OCT at the postoperative followup.

	23-gauge (microns) Average ± SD	25-gauge (microns) Average ± SD	*P* value
Postoperative day 1	1117 ± 247	977 ± 205	0.03
Postoperative day 7	1120 ± 234	944 ± 144	0.04
Postoperative day 30	1129 ± 254	944 ± 144	0.003

Total	1122 ± 242	974 ± 174	0.04

**Table 2:** Longitudinal length of sclerotomy incisions after transconjunctival sutureless pars plana vitrectomy performed with 23-gauge and 25-gauge measured with SD-OCT at the postoperative followup.

	23-gauge (microns) Average ± SD	25-gauge (microns) Average ± SD	*P* value
Postoperative day 1	375 ± 39	236 ± 18	<0.001
Postoperative day 7	357 ± 37	233 ± 23	<0.001
Postoperative day 30	355 ± 49	233 ± 23	<0.001

Total	363 ± 42	234 ± 19	<0.001

## References

[1] Fujii GY, De Juan E, Humayun MS (2002). A new 25-gauge instrument system for transconjunctival sutureless vitrectomy surgery. *Ophthalmology*.

[2] Fujii GY, De Juan E, Humayun MS (2002). Initial experience using the transconjunctival sutureless vitrectomy system for vitreoretinal surgery. *Ophthalmology*.

[3] Kunimoto DY, Kaiser RS (2007). Incidence of endophthalmitis after 20- and 25-gauge vitrectomy. *Ophthalmology*.

[4] Shaikh S, Ho S, Richmond PP, Olson JC, Barnes CD (2007). Untoward outcomes in 25-gauge versus 20-gauge vitreoretinal surgery. *Retina*.

[5] Scott IU, Flynn HW, Dev S (2008). Endophthalmitis after 25-gauge and 20-gauge pars plana vitrectomy: incidence and outcomes. *Retina*.

[6] Acar N, Kapran Z, Unver YB, Altan T, Ozdogan S (2008). Early postoperative hypotony after 25-gauge sutureless vitrectomy with straight incisions. *Retina*.

[7] Byeon SH, Lew YJ, Kim M, Kwon OW (2008). Wound leakage and hypotony after 25-gauge sutureless vitrectomy: factors affecting postoperative intraocular pressure. *Ophthalmic Surgery, Lasers & Imaging*.

[8] Gupta OP, Weichel ED, Regillo CD (2007). Postoperative complications associated with 25-gauge pars plana vitrectomy. *Ophthalmic Surgery Lasers and Imaging*.

[9] Kellner L, Wimpissinger B, Stolba U, Brannath W, Binder S (2007). 25-Gauge vs 20-gauge system for pars plana vitrectomy: a prospective randomised clinical trial. *British Journal of Ophthalmology*.

[10] Acar N, Kapran Z, Altan T, Unver YB, Yurtsever S, Kucuksumer Y (2008). Primary 25-gauge sutureless vitrectomy with oblique sclerotomies in pseudophakic retinal detachment. *Retina*.

[11] López-Guajardo L, Pareja-Esteban J, Teus-Guezala MA (2006). Oblique sclerotomy technique forprevention of incompetent wound closure in transconjunctival 25-gauge vitrectomy. *American Journal of Ophthalmology*.

[12] Inoue M, Shinoda K, Shinoda H, Kawamura R, Suzuki K, Ishida S (2007). Two-step oblique incision during 25-gauge vitrectomy reduces incidence of postoperative hypotony. *Clinical and Experimental Ophthalmology*.

[13] Shimada H, Nakashizuka H, Mori R, Mizutani Y, Hattori T (2006). 25-gauge scleral tunnel transconjunctival vitrectomy. *American Journal of Ophthalmology*.

[14] Singh RP, Bando H, Brasil OFM, Williams DR, Kaiser PK (2008). Evaluation of wound closure using different incision techniques with 23-gauge and 25-gauge microincision vitrectomy systems. *Retina*.

[15] Nagpal M, Wartikar S, Nagpal K (2009). Comparison of clinical outcomes and wound dynamics of sclerotomy ports of 20, 25, and 23 gauge vitrectomy. *Retina*.

[16] Yanyali A, Celik E, Horozoglu F, Nohutcu AF (2005). Corneal topographic changes after transconjunctival (25-gauge) sutureless vitrectomy. *American Journal of Ophthalmology*.

[17] Eckardt C (2005). Transconjunctival sutureless 23-gauge vitrectomy. *Retina*.

[18] Lott MN, Manning MH, Singh J, Zhang H, Singh H, Marcus DM (2008). 23-gauge vitrectomy in 100 eyes short-term visual outcomes and complications. *Retina*.

[19] Gupta OP, Ho AC, Kaiser PK (2008). Short-term outcomes of 23-gauge pars plana vitrectomy. *American Journal of Ophthalmology*.

[20] Parolini B, Romanelli F, Prigione G, Pertile G (2009). Incidence of endophthalmitis in a large series of 23-gauge and 20-gauge transconjunctival pars plana vitrectomy. *Graefe's Archive for Clinical and Experimental Ophthalmology*.

[21] Teixeira A, Allemann N, Yamada ACN, Uno F, Maia A, Bonomo PP (2009). Ultrasound biomicroscopy in recently postoperative 23-gauge transconjunctival vitrectomy sutureless self-sealing sclerotomy. *Retina*.

[22] Chen D, Lian Y, Cui L, Lu F, Ke Z, Song Z (2010). Sutureless vitrectomy incision architecture in the immediate postoperative period evaluated in vivo using optical coherence tomography. *Ophthalmology*.

[23] Taban M, Sharma S, Ventura AACM, Kaiser PK (2009). Evaluation of wound closure in oblique 23-gauge sutureless sclerotomies with visante optical coherence tomography. *American Journal of Ophthalmology*.

[24] Guthoff R, Riederle H, Meinhardt B, Goebel W (2010). Subclinical choroidal detachment at sclerotomy sites after 23-gauge vitrectomy: analysis by anterior segment optical coherence tomography. *Ophthalmologica*.

[25] Chen JC (1996). Sutureless pars plana vitrectomy through self-sealing sclerotomies. *Archives of Ophthalmology*.

[26] Kwok AKH, Tham CCY, Lam DSC, Li M, Chen JC (1999). Modified sutureless sclerotomies in pars plana vitrectomy. *American Journal of Ophthalmology*.

[27] Schmidt J, Nietgen GW, Brieden S (1999). Self-sealing, sutureless sclerotomy in pars-plana vitrectomy. *Klinische Monatsblatter fur Augenheilkunde*.

[28] Boker T, Spitznas M (1994). Ultrasound biomicroscopy for examination of the sclerotomy site after pars plana vitrectomy. *American Journal of Ophthalmology*.

[29] Rahman R, Rosen PH, Riddell C, Towler H (2000). Self-sealing sclerotomies for sutureless pars plana vitrectomy. *Ophthalmic Surgery and Lasers*.

[30] Taban M, Ventura AACM, Sharma S, Kaiser PK (2008). Dynamic evaluation of sutureless vitrectomy wounds: an optical coherence tomography and histopathology study. *Ophthalmology*.

